# Associations of Food Insecurity with Dietary Inflammatory Potential and Risk of Low Muscle Strength

**DOI:** 10.3390/nu15051120

**Published:** 2023-02-23

**Authors:** Su Min Kim, Yoon Jung Park, Hyesook Kim, Oran Kwon, Kwang Suk Ko, Yuri Kim, Yangha Kim, Hyesook Park, Seungyoun Jung

**Affiliations:** 1 Department of Nutritional Science and Food Management, Ewha Womans University, Seoul 03760, Republic of Korea; 2 Graduate Program in System Health Science and Engineering, Ewha Womans University, Seoul 03760, Republic of Korea; 3 Department of Food and Nutrition, Wonkwang University, 460, Iksan-daero, Iksan-si 54538, Republic of Korea; 4 Department of Preventive Medicine, College of Medicine, Ewha Womans University, Seoul 03760, Republic of Korea

**Keywords:** food insecurity, dietary inflammatory index, muscle strength, hand grip strength, inflammation

## Abstract

Food insecurity refers to the uncertain availability of or limited access to nutritious food. Poor diets prevalent among food insecure populations may incite an inflammatory state and subsequently negatively affect skeletal muscle metabolism. To examine the inflammatory mechanistic potential of the association between food insecurity and the risk of low muscle strength, we analyzed cross-sectional data from 8624 adults aged ≥20 years from the Korean National Health and Nutrition Examination Survey 2014–2015. Household food security status was assessed using an 18-item food security survey module. The inflammatory potential of diets was estimated by the dietary inflammation index (DII). Low muscle strength was ascertained using hand grip strength. In the multivariable-adjusted model, greater food insecurity was significantly associated with a higher DII score and risk of low muscle strength. The multivariable-adjusted mean difference (95% confidence interval) on the DII, comparing the “moderate-to-severe” food insecurity group with the “food secure” group, was 0.43 (0.06–0.80) (P-trend: <0.001) and the odds ratio (95% confidence intervals) of low muscle strength for the same comparison groups was 2.06 (1.07–3.96) (P-trend: 0.005). Our results suggest that individuals with greater food insecurity may be susceptible to diets with greater inflammatory potential, which may contribute to a loss of muscle strength.

## 1. Introduction

Skeletal muscle is an integral body tissue that plays a pivotal role in physical strength, physical performance, and metabolic regulation [1]. Muscle strength is an indicator of muscle function and is increasingly seen as a robust predictor of a range of health outcomes [2,3,4,5]. For instance, hand grip strength (HGS), a simple and reliable measurement of muscle strength [6,7], has been inversely associated with the risk of physical disability, respiratory disease, cancers, cardiovascular diseases, and all-cause and cardiovascular mortality [2,8]. The onset of loss of muscle mass and muscle strength begins in the early 30s and accelerates with aging, being observed to disproportionally affect individuals of low socioeconomic status [9,10].

Food insecurity refers to a lack of consistent access to sufficient, safe, and nutritious food for an active, healthy life and is associated with poor nutritional status [11] and unfavorable health outcomes, including obesity, metabolic syndrome, and diabetes [12]. According to the United Nations, nearly 2.3 billion people, accounting for 29.3% of the global population, were reported to be moderately or severely food insecure in 2021, which is an increase of 350 million since the onset of the COVID-19 pandemic [13]. Diet is well known to be important for preserving healthy muscle [14,15], and individuals at risk of food insecurity are thus more likely to be susceptible to muscle deterioration. Indeed, the positive association of food insecurity with the risk of low muscle strength has been reported in two previous cross-sectional studies [16,17], but evidence is still limited [16,17] and the underlying mechanisms of the associations have not been elucidated. 

Nevertheless, substantial evidence suggests that food insecurity may affect muscle strength by altering the inflammatory state of the body. Diet is thought to modulate systemic inflammation, whose association with inflammatory biomarkers is well-documented in the literature [18], and inflammation is increasingly recognized as having a negative effect on muscle maintenance, altering cellular protein metabolisms to favor proteolysis over synthesis [19,20,21,22]. Previously reported associations of a pro-inflammatory diet [23,24,25] or inflammatory markers [22,26,27] with the risk of low muscle strength further support this speculation. However, there is a paucity of evidence that the poor diets prevalent among food insecure groups indeed incite diets with high inflammatory potential [28,29].

Therefore, we hypothesized that the greater possibility of inflammation via poor diets associated with food insecurity may have a significant implication for muscle strength. To examine our hypothesis, we investigated (1) the association between food insecurity levels and the dietary inflammatory index (DII), a well-known composite measure for estimating the inflammatory capacity of the entire diet [18], and (2) the association between food insecurity and the risk of low muscle strength in the Korea Health and Nutrition Survey (KNHANES), a nationally representative cross-sectional survey of Koreans [30]. In a secondary analysis, we also explored whether the association of low muscle strength with levels of food insecurity varies according to population characteristics. 

## 2. Materials and Methods

### 2.1. Study Population

This study used data from the 6th KNHANES (2104–2015). In brief, the KNHANES is an ongoing national survey that has been conducted by the Korea Centers for Disease Control (KCDC) since 1998 [30]. As a nationwide survey, the KNHANES enrolls nationally representative non-institutional civilians following a multi-level clustered probabilistic sampling design. The KNHANES consists of a household screening survey, health interview, health examination, and nutritional survey and collects details of several variables pertaining to the demographic, social, health, and nutritional status of the participants. All surveys are conducted with the consent of the participants. The institutional review boards of the KCDC have approved all KNHANES protocols. The present study was exempt from review by the institutional review board of Ewha Womans University because it used de-identified and publicly available data (IRB no. 202209-0020-01).

**Figure 1:**
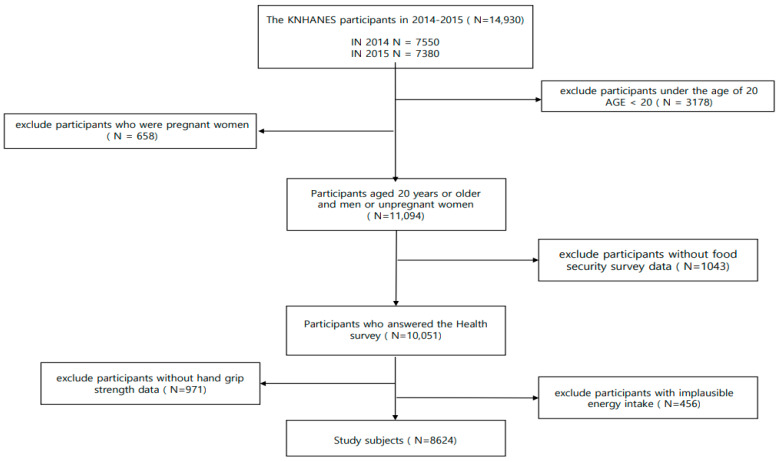
Flow diagram of the study population selection.

Of the 14,930 individuals who participated in the KNHANES (2014–2015), we excluded those who: (1) were under 19 years of age (N = 3178), (2) were pregnant (N = 658), (3) provided no response to the food security survey (N = 1043), (4) had no measurements for HGS (N = 971), and (5) had implausible energy intake (≤500 kcal, ≥5000 kcal [31]) (N = 456) (Figure 1). Consequently, the final sample size analyzed in our study was 8624 adults. 

### 2.2. Assessment of Food Insecurity 

The primary exposure of interest was the household food security level. Household food insecurity was measured using the revised 18-item US Household Food Security Survey Module (USHFSSM) [32], the use of which has previously been validated in the Korean population [33]. In brief, the USHFSSM asks questions related to household- and individual-level experiences or behaviors relating to food purchasing, food availability, and diet over the previous 12 months. Of the 18 questions, eight pertain to children’s experiences and thus were omitted if no child was present in the household. The overall level of food insecurity was quantified by scoring the responses to the USHFSSM. Applying the USDA standard coding procedure of giving a point to affirmative responses indicative of insufficient resources for food, 0 or 1 points were allocated to each question [32]. For example, 1 point was given to those who answered “yes” to questions with two responses (“yes” or “no”) or those who answered “frequently” or “occasionally” to questions with three responses (“frequently”, “occasionally”, or “never”). The points from all 18 questions were summed to create a food insecurity score within the range of 0–18 for households with children and 0–10 for households without children. Higher scores indicate greater food insecurity levels. The reliability coefficient α of the USHFSSM is ≥0.89 for households both with and without children, which suggests a high level of confidence [31]. 

The USDA guidelines define food insecurity levels into four categories: “food secure” (score of 0–2 for households with/without children), “food insecure without hunger” (3–7 for households with children; 3–5 for households without children), “moderately food insecure with hunger” (8–12 for households with children; 6–8 for households without children), and “severely food insecure with hunger” (13–18 for households with children; 9–10 for households without children) [32]. However, the number of individuals within the “moderately food insecure with hunger” and “severely food insecure with hunger” categories was small in our study. Thus, we combined the latter two USDA categories and analyzed our study population by re-defining the food insecurity level into three groups, as follows [34]: “food secure” (USDA food secure group), “mildly food insecure” (USDA food insecure without hunger group), and “moderate-to-severe food insecurity” (USDA moderately and severely food insecure with hunger groups). 

### 2.3. Measurement of Dietary Inflammatory Potential

The inflammatory potential of diets was assessed using the DII, which was developed by Shivappa et al. [18]; the details of its development [18] and validation [35,36] have been given previously. In brief, the DII is a composite score of 45 food parameters, including micronutrients, macronutrients, and individual food items, weighted by their dietary inflammatory effects. Each food parameter’s inflammatory effect was quantified as a score on the basis of its associations with inflammatory markers obtained from nearly 2000 articles [18]. To calculate the DII, the present study used DII food parameter intake data obtained from 24 h recall. The following 23 of the 45 DII food parameters in the original DII scoring algorithm were available and used for the DII calculation: energy, vitamin A, vitamin C, carbohydrates, proteins, total fat, saturated fatty acids, single unsaturated fatty acids, polyunsaturated fatty acids, omega-3 fatty acids, omega-6 fatty acids, cholesterol, dietary fiber, iron, carotene, thiamine, riboflavin, niacin, garlic, onions, ginger, pepper, and tea (black tea, green tea). For each DII food parameter, the actual intake was first standardized to a z-score using the world standard mean and deviation derived from the world composite database from 11 nations [18]. The z-score was then converted into a percentile and centered on zero by doubling the value and subtracting 1. The centered percentile intake value was multiplied by the respective inflammatory effect score to create a food-parameter-specific DII score. Finally, the DII score for each food parameter was summed across all the available food parameters to create an overall DII. A higher DII score suggests a higher pro-inflammatory potential for the diet. 

### 2.4. Ascertainment of Low Muscle Strength 

Muscle strength was estimated by measuring HGS, a well-known and reliable measure of muscle strength [6,7], using a digital grip strength dynamometer (Model T.K.K.5401, Takei Co., Ltd., Ishioka, Japan). The HGS testing was performed according to the standardized manual recommended by the Centers for Disease Control and Prevention, and the dynamometer was calibrated and checked by trained staffs to ensure the accuracy of the data [37]. HGS was assessed in a standing position with the arm and wrist in the anatomical position, and participants were asked not to wield the grip dynamometer or hold their breath during testing [38]. HGS was measured three times on each hand with a 1 min interval between each trial, alternating the left and right hands. To determine low muscle strength, the maximally measured HGS value out of the three measurements of the dominant hand was used, and low muscle strength was defined as <28 kg for men and <18 kg for women, using the recently revised diagnostic criteria recommended by the Asian Working Group for Sarcopenia [39,40]. 

### 2.5. Assessment of Covariates

The KNHANES collected data on sociodemographics (age, sex, marital status, area of residence, education, income), behavioral factors (body mass index (BMI), smoking, alcohol drinking, activity), and health status using standard self-reported questionnaires [30]. Using the information from these questionnaires, residential areas were defined as either urban or rural, according to the addresses of the participants. Smoking and alcohol drinking status were defined as never, past, or current. Physical activity was assessed through the International Physical Activity Questionnaire (IPAQ) [41,42]; participants were defined as having low, medium, or high levels of physical activity if their activity level was <600 metabolic equivalent (MET) minutes per week (min/week), 600–3000 MET (min/week), or ≥3000 MET (min/week) [41]. Height and weight were measured by trained staff, and BMI was calculated as the square (kg/m^2^) of weight (kg) over height (m). Comorbidities were defined by counting the number of existing chronic disease conditions, including diabetes mellitus, hypertension, dyslipidemia, stroke, myocardial infarction, angina pectoris, pulmonary tuberculosis, tuberculosis, and asthma [24,43]. Dietary data were collected via 24 h recall with the assistance of trained interviewers and converted to individual nutrient intakes based on the national standard food composition table [44]. 

## 3. Statistical Analyses

Sampling weights were applied to all analyses to yield unbiased national estimates of the KNHANES. The characteristics of the study population according to the food security level were averaged ± standard deviation using the SURVEYMEANS procedure for continuous variables, and the categorical variables were measured as numbers and percentages using the SURVEYFREQ procedure. 

The analysis evaluated the association of food security with the DII score using the SURVEYREG procedure, while the association between food security and low muscle strength was evaluated using the SURVEYLOGISTIC procedure. To address the potential for confounding, well-known risk factors of muscle strength and food security were selected *a priori* from previous studies [16,17] and adjusted in the multivariable model as follows: age (continuous, year), sex (male, female), marital status (married, never married), residence (urban, rural), education level (less than elementary school, middle school graduate, high school graduate, college graduate or higher, missing), income (quartiles, missing), BMI (underweight, normal, overweight, obese, missing), smoking status (never, past, current, missing), alcohol drinking status (never, past, current), physical activity (high, medium, low), and number of chronic disease (0, 1, ≥2, missing). An indicator for missing responses in the covariates was created, if applicable. The P-trend was tested by modeling the median value for each food insecurity category as a continuous term. We also conducted several sensitivity analyses, defining low muscle strength by using age-specific criteria to take into account the variability of muscle strength with aging [45] and including protein intake, which was primarily not adjusted for because it could mediate the association between food insecurity and muscle strength. To examine whether associations varied by population characteristics, stratified analyses were conducted by sex, age, physical activity level, BMI, and smoking status. P for interaction was tested using the cross-product term between food security levels and stratification factors. 

All analyses were performed using SAS version 9.4 (SAS Institute, Inc., Cary, NC, USA). All statistical tests were two-sided at a significance level <0.05. 

## 4. Results

The characteristics of the study population are described in Table 1. Of the 8624 Korean adults in our study, 7995 (92.4%) were food secure, 529 (6.4%) were mildly food insecure, and 100 (1.2%) were moderate-to-severe food insecure. The mean age of our study population was 46.6 ± 0.3 years, and 50.8% were male. The majority were married (78.5%), never smokers (54.1%), and current alcoholic drinkers (73.0%) and lived in rural regions (69.7%), engaged in a low level of physical activity (63.8%), and had no chronic disease condition (75.3%). Participants with a greater level of food insecurity were more likely to be older, have less than college or higher education, earn a lower income, be overweight, and currently smoke, while also drinking less alcohol, being less physically active, and having a greater number of chronic disease conditions. They were also more likely to have greater DII and lower HGS levels (Supplementary Table S1) Sex, marital status, and residence were similar regardless of food security levels.

Table 2 shows the association between food insecurity and the DII score. In our analyses estimating the mean difference in DII score when comparing groups with increasing levels of food insecurity, we observed a significant positive dose-response trend for the association between food insecurity and DII score in both the age-adjusted and multivariable-adjusted models. Compared to the food secure group, the mean DII (95% CI) was higher by 0.36 (0.21–0.51) for the mildly food insecure group and by 0.69 (0.28–1.10) for the moderate-to-severe food insecure group in an age-adjusted model (P-trend 0.01). Similarly, the mean DII (95% CI) was higher by 0.15 (0.01–0.30) for the mildly food insecure group and 0.43 (0.06–0.80) for the moderate-to-severe food insecure group compared to the food secure group in the multivariable-adjusted model (P-trend < 0.001). 

Table 3 shows the results for the association between food insecurity and the risk of low muscle strength. We observed a statistically significant positive dose-response trend for the association between food insecurity and the risk of low muscle strength in both age-adjusted and multivariable-adjusted models. The age-adjusted OR (95% CI) compared to the food secure group was 1.38 (0.98–1.95) for the mildly food insecure group and 2.76 (1.55–4.92) for the moderate-to-severe food insecure group (P-trend: <0.001). The multivariable-adjusted OR (95% CI) compared with the food secure group was 1.18 (0.83–1.70) for the mildly food insecure group and 2.06 (1.07–3.96) for the moderate-to-severe food insecure group (P-trend: 0.005). In our sensitivity analyses to test the robustness of our results, the inverse associations did not change substantially when protein intakes were further adjusted in the model (Supplementary Table S2) or when an age-specific cut-off was used to define low muscle strength [45] (Supplementary Table S3).

We further explored possible effect modification by population characteristics for the association between food insecurity and the risk of low muscle strength (Table 4). However, the positive association observed between food insecurity and the risk of low muscle strength was not significantly modified by sex, age, physical activity, BMI, or smoking status (all P-interaction ≥ 0.43). 

## 5. Discussion

In this nationally representative cross-sectional study of Korean adults, greater food insecurity was associated with diets with greater inflammatory potential, as measured by the DII, and an elevated risk of low muscle strength. The positive association of food insecurity with risk of low muscle strength did not significantly vary by sex, age, BMI, smoking status, or physical activity. Given the increased risk of morbidity and mortality associated with weak muscle strength [2,3,4,5], the identification of individuals at risk of muscle loss and its underlying mechanisms is important. Our results reinforce findings on the adverse health impact of food insecurity [12] and further expand the literature suggesting greater susceptibility to pro-inflammatory processes and muscle decline among individuals with lower food security.

Food insecurity refers to limited or uncertain access to or availability of nutritionally adequate and safe foods [12], which may lead to suboptimal, inadequate diets and compensatory reliance on affordable low-cost energy-dense foods. The increased risk of poor diet among food insecure groups is well established [11,34,46]. However, there are few studies on the inflammatory potential of the diet consumed by food insecure groups [28,29], which may underlie the poor health outcomes associated with food insecurity [12]. To date, two cross-sectional studies have examined the association between food insecurity and the DII [28,29]. A previous study of 10,630 low-income adults in the US reported a greater DII score, by 0.31, among the very low food secure group compared to the high food secure group (*p* = 0.0033) [28]. Similarly, results from 525 high school girls in Iran showed a greater DII score, by 0.025, for every one unit increase in the food insecurity score [29]. A recent Hungarian study also observed a greater DII level among the Hungarian Rome region population at risk of greater food insecurity [47] than the general population [48]. Our positive association of food insecurity with the DII adds to the consistent, though limited, literature. 

Previous studies that examined the intake of individual nutrients [11,49] or diet patterns [11,34] in relation to food insecurity further support our positive association of food insecurity with the DII. A systematic review of 26 previous studies [11] and subsequent large national representative studies in the US [50], Canada [50], Brazil [51], and Korea [34,49] consistently reported that food insecure adults adhere less to a healthy diet and consume fewer vegetables and fruits and less fiber and polyunsaturated fat, but have a greater intake of calories, sugars, and saturated fat, which are associated with pro-inflammation [18,52]. This evidence thus indicates that a lack of food availability and disturbed access to food may induce an adverse change in diet quality, which may translate into a diet with greater inflammatory potential, thereby explaining our results, and predispose individuals to greater risk of chronic inflammation. 

An altered inflammatory state has been implicated in many chronic diseases, including metabolic syndrome, type 2 diabetes, cardiovascular diseases, and various cancers [53], but relatively little attention has been paid to muscle strength. However, proinflammatory cytokines have been speculated to contribute to skeletal muscle loss [21] by impairing insulin signaling associated with protein synthesis [54] through the transcriptional activation of NF-kB or the induction of JK that contributes to phosphorylation of serine residue in IRS-1 [55]. Indeed, substantial evidence from animal and human studies shows that greater inflammatory milieu negatively affects muscle protein metabolism and skeletal muscle maintenance [19,21,22]. For example, in experimental studies, rats administered IL-6 or TNF-α were shown to have elevated skeletal muscle protein breakdown, decreased protein synthesis, and reduced total skeletal muscle amino acid concentration [56,57]. Similarly, a systematic review and meta-analysis of 149 cross-sectional studies and 19 longitudinal studies reported associations between higher levels of C-reactive protein (CRP), IL-6, and TNF-α and decreasing muscle strength [46]. These results were further confirmed in subsequent large, national, cross-sectional studies of 8232 adults [22], 2127 postmenopausal women [27], and 917 adolescents [26], which found significantly lower HGS with increasing inflammatory biomarker profiles, such as white blood cell counts [26], CRP [27], and systemic immune-inflammation index scores, which combine platelets, neutrophils, and lymphocytes [22]. 

Thus, food insecurity associated with a poor diet with greater inflammatory potential may accelerate degradation of muscle strength partly through the inflammatory pathway. Indeed, the majority of the previous four cross-sectional studies that evaluated the associations of DII with the risk of low muscle strength showed lower HGS among those with greater DII [23,24,25], although not all [58]. However, few studies have precisely evaluated the association between food insecurity and the risk of low muscle strength [16,17]. Our results, which indicate a positive association between food insecurity and the risk of low HGS, contribute to this limited literature, aligning with three previous studies’ results [16,17,59]. In the first, a National Health and Nutrition Examination Surveys study of the elderly (*n* = 3632) revealed a significantly increased association between food insecurity and risk of low HGS (OR:1.51, 95% CI: 1.03–2.22) [16]. In the second, a cross-sectional analysis of 664 Iranian adults, the odds ratios for food insecurity in men and women with low HGS were 5.46 and 3.12, respectively (*p* < 0.001) [17]. In the third, the Global Aging and Adult Health study of 14,585 adults also showed a significant 2.05 times increased risk of sarcopenia, defined as low muscle mass and either low muscle strength or slow gait [59].

Our study has several strengths. It is the first study to evaluate the plausibility of an inflammatory mechanism possibly mediated by diet through the association between food insecurity and muscle strength. Household food insecurity levels were measured using the most accurate instrument, that is, the full 18 items of the USHFSSM [32], the reliability and validity of which have previously been demonstrated in Korea [33]. Across the wide range of the USHFSSM’s food insecurity scale, we were able to evaluate the dose-response associations of food insecurity with diet and the risk of low muscle strength for various levels of food insecurity. Moreover, the quality of our HGS data was high given the multiple measurements of HGS performed by trained staff following the KCDC’s standardized HGS test protocols. We also used the most up-to-date diagnostic criteria to define low muscle strength in Asians, which account for the relatively small body size and adiposity compared to Caucasians [39]. Using the DII, we examined the possible impact of food insecurity across the overall inflammatory potential of the diet, accounting for complex interactions of nutrients beyond individual nutrients or food items [60]. Finally, utilizing data on food security, grip strength, diet, demographics, medical conditions, and lifestyle from the KNHANES, we comprehensively adjusted for the known possible confounding factors of low muscle strength and food insecurity and tested the robustness of our results by conducting several subgroup and sensitivity analyses.

There are several limitations to our study. First, the cross-sectional nature of our study design makes it difficult to determine the temporality between food insecurity and muscle strength data, which limits the causal interpretations of our results. Our inverse association between food insecurity and the risk of muscle strength can be alternatively interpreted as the consequence of low muscle strength limiting individuals’ behaviors and worsening their food insecurity. Second, although the use of 24 h recall in national nutritional surveys is common and has proven validity, precision, and high response rates [61], our 24 h recall dietary data are susceptible to random measurement error when estimating usual diets due to the inability to capture the day-to-day variations in food consumption. We calculated the DII using only 23 out of the 45 food parameters that comprise the DII due to the lack of dietary data in the KNHANES. Nonetheless, missing food parameters such as turmeric and saffron are likely to make up only a small proportion of total nutrients consumed by the general population. Furthermore, the significant association of the DII with circulating levels of inflammatory markers observed in previous studies with a limited number of food parameters, as in the present study, validates the use of the DII without the full 45 food parameters [25]. Finally, despite our comprehensive adjustment of potential confounding factors, we cannot rule out the possibility of unknown or residual confounding variables. 

## 6. Conclusions

In conclusion, food insecurity was associated with diets with a greater DII score and an increased risk of low muscle strength in this nationally representative adult population in Korea. These findings support the notion that inflammation is likely to be elevated among those at risk of food insecurity, in part due to their nutritiously poor, pro-inflammatory diets, which may negatively affect muscle maintenance and muscle strength. Further large cohort studies confirming our results are warranted and will assist in generating useful information to support the design of evidence-based nutritional strategies to reduce the health burden among populations at risk of food insecurity. 

## Figures and Tables

**Table 1:** Study population characteristics ^1^ (N = 8624).

		Food Insecurity Level		
Characteristics	All	Food Secure (N = 7995)	Mildly Food Insecure (N = 529)	Moderate-to-Severe Food Insecure (N = 100)
**Sociodemographic factors**				
Age, years	46.6 ± (0.3)	46.5 ± (0.3)	48.4 ± (1.0)	49.1 ± (2.1)
Sex				
Male	3675 (50.8)	3424 (51.0)	210 (47.8)	41 (52.0)
Female	4949 (49.2)	4571 (49.0)	319 (52.2)	59 (48.0)
Marital status				
Married	7434 (78.5)	6900 (78.6)	448 (77.9)	86 (79.3)
Never married	1190 (21.5)	1095 (21.4)	81 (22.1)	14 (20.7)
Residence				
Urban	2479 (30.3)	2294 (30.4)	157 (29.6)	28 (31.0)
Rural	6145 (69.7)	5701 (69.6)	372 (70.4)	72 (69.0)
Education level				
Less than elementary school	1861 (14.1)	1638 (13.2)	185 (24.5)	38 (26.3)
Middle school graduate	879 (8.3)	802 (8.0)	61 (11.6)	16 (16.0)
High school graduate	2523 (33.2)	2350 (33.1)	153 (35.9)	20 (25.6)
College graduate or higher	2596 (35.4)	2501 (36.7)	86 (19.6)	9 (14.3)
Missing	765 (9.1)	704 (9.0)	44 (8.5)	17 (18.0)
Income				
Quartile 1	2073 (24.4)	1752 (22.2)	255 (49.5)	66 (71.0)
Quartile 2	2142 (24.6)	1960 (24.3)	157 (30.2)	25 (20.7)
Quartile 3	2164 (24.6)	2081 (25.6)	77 (13.2)	6 (5.8)
Quartile 4	2206 (25.9)	2169 (27.4)	35 (6.3)	2 (1.1)
Missing	39 (0.5)	33 (0.5)	5 (0.8)	1 (1.3)
**Behavioral factors**				
BMI (kg/m^2^)				
Underweight	331 (4.1)	299 (4.0)	26 (4.6)	6 (6.6)
Normal	3282 (38.5)	3057 (38.6)	193 (37.8)	32 (30.8)
Overweight	2037 (23.0)	1889 (23.1)	120 (21.0)	28 (29.9)
Obese	2962 (34.3)	2738 (34.2)	190 (36.7)	34 (32.7)
Missing	12 (0.1)	12 (0.1)	0	0
Smoking status				
Never	5051 (54.0)	4716 (54.4)	286 (49.5)	49 (50.5)
Past	1739 (20.2)	1620 (20.4)	100 (19.4)	19 (14.2)
Current	1416 (21.1)	1283 (20.7)	110 (26.3)	23 (27.4)
Missing	418 (4.6)	376 (4.5)	33 (4.9)	9 (8.0)
Alcohol drinking status				
Never	1034 (8.8)	949 (8.6)	74 (11.5)	11 (7.0)
Past	1374 (13.7)	1239 (13.4)	111 (17.3)	24 (22.7)
Current	5806 (73.0)	5437 (73.5)	313 (66.5)	56 (62.3)
Missing	410 (4.5)	370 (4.5)	31 (4.7)	9 (8.0)
Physical activity ^2^				
High	28 (0.4)	26 (0.5)	1 (0.08)	1 (1.1)
Medium	2965 (35.8)	2772 (36.2)	164 (31.1)	29 (28.3)
Low	5631 (63.8)	5197 (63.4)	364(68.9)	70 (70.6)
**Clinical health conditions**				
The number of chronic disease ^3^				
0	5835 (75.3)	5445 (75.7)	329 (69.8)	61 (71.4)
1	1568 (14.2)	1431 (14.0)	115 (18.1)	22 (13.4)
≥2	1036 (8.3)	946 (8.1)	13 (11.2)	13 (11.2)
Missing	185 (2.1)	173 (2.1)	4 (4.0)	4 (4.0)

Abbreviations: BMI, body mass index. ^1^ Data are expressed as means ± (s.d.) for continuous variables or frequency (percentages) for categorical variables, or otherwise specified. Statistics are weighted using the individual weights provided by the KNHANES study participants. ^2^ Physical activity was defined to be high if the activity level was >3000 MET-min/wk, medium if the activity level was >600–≤3000 MET-min/wk, and low if the activity level was ≤600 MET-min/wk. ^3^ Numbers of chronic diseases were calculated by counting the diabetes mellitus, hypertension, dyslipidemia, stroke, myocardial infarction angina pectoris, pulmonary tuberculosis, and asthma.

**Table 2:** Age-adjusted and multivariable-adjusted ^1^ mean differences of dietary inflammatory index (DII) (95% confidence interval) according to food insecurity levels.

	Food Insecurity Level			P-Trend ^2^
	Food Secure (Referent)	Mildly Food Insecure	Moderate-to-Severe Food Insecure	
Age-adjusted DII mean difference (95% CI)	0.00 (ref)	0.36 (0.21–0.51)	0.69 (0.28–1.10)	0.01
MV-adjusted DII mean difference (95% CI)	0.00 (ref)	0.15 (0.01–0.30)	0.43 (0.06–0.80)	<0.001

^1^ Multivariable model adjusted for age (continuous, year), sex (male, female), marital status (married, never married), residence (urban, rural), education level (less then elementary school, middle school graduate, high school graduate, college graduate or higher, missing), income (quartiles, missing), BMI (underweight, normal, overweight, obese, missing), smoking status (never, past, current, missing), alcohol drinking status (never, past, current), physical activity (high, medium, low), and number of chronic disease (0, 1, ≥2, missing). ^2^ P-trend was calculated using the median value of each food insecurity category as a continuous variable.

**Table 3:** Age- and multivariable-adjusted ^1^ odds ratios (95% confidence intervals) of low muscle strength according to food insecurity levels.

	Food Insecurity Level			P-Trend ^2^
	Food Secure (Referent)	Mildly Food Insecure	Moderate-to-Severe Food Insecure	
No. cases/No. non-cases	622/7373	67/462	19/81	
Age-adjusted OR (95% CI)	1.00 (ref)	1.38 (0.98–1.95)	2.76 (1.55–4.92)	<0.001
MV-adjusted OR (95% CI)	1.00 (ref)	1.18 (0.83–1.70)	2.06 (1.07–3.96)	0.005

^1^ Multivariable model adjusted for age (continuous, year), sex (male, female), marital status (married, never married), residence (urban, rural), education level (less then elementary school, middle school graduate, high school graduate, college graduate or higher, missing), income (quartiles, missing), BMI (underweight, normal, overweight, obese, missing), smoking status (never, past, current, missing), alcohol drinking status (never, past, current), physical activity (high, medium, low), and number of chronic disease (0, 1, ≥2, missing). ^2^ P-trend was calculated using the median value of each food insecurity category as a continuous variable.

**Table 4:** Multivariable-adjusted ^1^ odds ratios (95% confidence intervals) of low muscle strength according to food insecurity levels by population characteristics.

	No. Cases /No. Non-Cases	Food Insecurity Levels			P-Trend ^2^	P-Interaction ^3^
		Food Secure (Reference)	Mildly Food Insecure OR (95% CI)	Moderate-to-Severe Food Insecure OR (95% CI)		
**Sex**						
Male	251/3424	1.00 (ref)	1.19 (0.77–1.84)	1.64 (0.71–3.8)	0.03	0.50
Female	457/4492	1.00 (ref)	1.12 (0.73–1.72)	1.47 (0.68–3.14)	0.08	
**Age**						
≥60 years	129/5519	1.00 (ref)	1.31 (0.74–2.33)	1.36 (0.39–4.77)	0.21	0.84
<60 years	579/2397	1.00 (ref)	1.11 (0.75–1.63)	2.22 (1.05–4.66)	0.08	
**Physical activity ^4^**						
≥ Medium	156/2837	1.00 (ref)	1.88 (0.98–3.62)	2.34 (0.84–6.52)	0.98	0.87
Low	552/5079	1.00 (ref)	1.03 (0.71–1.50)	0.91 (1.03–3.54)	0.04	
**BMI**						
≥25 (kg/m^2^)	491/2640	1.00 (ref)	1.20 (0.64–2.26)	2.62 (0.98–7.01)	0.10	0.64
<25 (kg/m^2^)	217/5276	1.00 (ref)	0.97 (0.49–1.90)	3.36 (1.10–10.27)	0.03	
**Smoking status**						
No current	564/6245	1.00 (ref)	1.08 (0.74–1.58)	1.48 (0.81–2.67)	0.06	0.43
Current	51/1346	1.00 (ref)	1.50 (0.5–4.43)	4.71 (1.23–18.1)	0.005	

^1^ Multivariable model adjusted for age (continuous, year), sex (male, female), marital status (married, never married), residence (urban, rural), education level (less then elementary school, middle school graduate, high school graduate, college graduate or higher, missing), income (quartiles, missing), BMI (underweight, normal, overweight, obese, missing), smoking status (never, past, current, missing), alcohol drinking status (never, past, current), physical activity (high, medium, low), and number of chronic disease (0, 1, ≥2, missing). ^2^ P-trend was calculated using the median value of each food insecurity category as a continuous variable. ^3^ P for interaction was tested using the cross-product term between food security levels and stratification factors. ^4^ Physical activity was defined to be greater than or equal to medium if the activity level was >600 MET-min/week and low if the activity level was ≤600 MET-min/week.

## Data Availability

Data described in the article and codebook are publicly and freely available without restriction at https://knhanes.kdca.go.kr/knhanes/main.do (accessed on 1 September 2020).

## Supplementary Materials

The following supporting information can be downloaded at: https://www.mdpi.com/article/10.3390/nu15051120/s1, Table S1: Median and interquartile range of DII and hand group strength according to food insecurity level; Table S2: Age- and multivariable-adjusted odds ratios (95% confidence intervals) of low muscle strength according to food insecurity levels additionally including protein intakes; Table S3: Age- and multivariable-adjusted odds ratios (95% confidence intervals) of low muscle strength according to food insecurity levels using an age-specific cut-off to define low muscle strength [45].

## Abbreviations

BMI: body mass index; DII, dietary inflammatory index.
