# Supplementary material for: Associations of Food Insecurity with Dietary Inflammatory Potential and Risk of Low Muscle Strength
Source: Nutrients. 2023 Feb 23;15(5):1120. doi: 10.3390/nu15051120 (PMC10005676; doi:10.3390/nu15051120)
Supplement: Supplementary file 1 [file nutrients-15-01120-s001.zip › nutrients-2226739-supplementary.pdf]

Supplementary Table S1. Median and interquartile range of DII and hand group strength according to food insecurity levels

Outcome measures	All	Food insecurity level		
		Food secure (N=7,995)	Mildly food insecure (N=529)	Moderate-to-severe food insecure (N=100)
DII	1.08 (0.10-2.15)	1.05 (0.09-2.11)	1.52 (0.41-2.65)	2.04 (0.30-3.00)
Hand Grip Strength	32.19 (25.51-42.62)	32.39 (25.65-42.80)	30.45 (23.88-40.76)	28.82 (22.07-39.78)

Abbreviations: DII, dietary inflammatory index

Supplementary Table S2. Age- and multivariable[1] adjusted odds ratio (95% confidence intervals) of low muscle strength according to food insecurity levels additionally including protein intakes

	Food insecurity level			P-trend[2]
	Food secure	Mildly food insecure	Moderate-to-severe food insecure	
N. cases/ N. non cases	622/7,373	67/462	19/81	
Age-adjusted OR (95% CI)	1.00 (ref)	1.38 (0.98-1.95)	2.76 (1.55-4.92)	<.001
MV-adjusted OR (95% CI)	1.00 (ref)	1.17 (0.82-1.69)	2.01 (1.04-3.87)	0.01

[1] Multivariable model adjusted for age (continuous, year), sex (male, female), marital status (married, never married), residence (urban, rural), education level (less then elementary school, middle school graduate, high school graduate, college graduate or higher, missing), income (quartiles, missing), bmi (underweight, normal, overweight, obese, missing), smoking status (never, past, current, missing), alcohol drinking status (never, past, current), physical activity (high, medium, low), number of chronic disease (0, 1, ≥2, missing), and protein intakes (continuous, g/d).

[2] P-trend was calculated using the median value of each food insecurity category as a continuous variable

Supplementary Table S3. Age- and multivariable[1] adjusted odds ratio (95% confidence intervals) of low muscle strength according to food insecurity levels using age-specific cut-off to define low muscle strength[2]

	Food insecurity level			P-trend[3]
	Food secure	Mildly food insecure	Moderate-to-severe food insecure	
N. cases/ N. non cases	509/7,486	58/471	18/82	
Age-adjusted OR (95% CI)	1.00 (ref)	1.34 (0.94-1.93)	2.91 (1.59-5.35)	<.001
MV-adjusted OR (95% CI)	1.00 (ref)	1.15 (0.78-1.70)	2.41 (1.18-4.90)	0.006

[1] Multivariable model adjusted for age (continuous, year), sex (male, female), marital status (married, never married), residence (urban, rural), education level (less then elementary school, middle school graduate, high school graduate, college graduate or higher, missing), income (quartiles, missing), bmi (underweight, normal, overweight, obese, missing), smoking status (never, past, current, missing), alcohol drinking status (never, past, current), physical activity (high, medium, low), and number of chronic disease (0, 1, ≥2, missing).

[2] Low muscle strength was defined if participants meet the following age-specific cut-off: <29.6 kg for men and 16.8 kg for women <28 kg if aged 20-39 years and 28.6 kg for men and 16.4 kg for women if aged >39 years [45]

[3] P-trend was calculated using the median value of each food insecurity category as a continuous variable
